# Multi-objective Optimization for Materials Discovery via Adaptive Design

**DOI:** 10.1038/s41598-018-21936-3

**Published:** 2018-02-27

**Authors:** Abhijith M. Gopakumar, Prasanna V. Balachandran, Dezhen Xue, James E. Gubernatis, Turab Lookman

**Affiliations:** 1 Los Alamos National Laboratory, Theoretical Division, Los Alamos, 87545 USA; 2 State Key Laboratory for Mechanical Behavior of Materials, Xian Jiaotong University, Xian, 710049 China

## Abstract

Guiding experiments to find materials with targeted properties is a crucial aspect of materials discovery and design, and typically multiple properties, which often compete, are involved. In the case of two properties, new compounds are sought that will provide improvement to existing data points lying on the Pareto front (PF) in as few experiments or calculations as possible. Here we address this problem by using the concept and methods of optimal learning to determine their suitability and performance on three materials data sets; an experimental data set of over 100 shape memory alloys, a data set of 223 *M*_2_*AX* phases obtained from density functional theory calculations, and a computational data set of 704 piezoelectric compounds. We show that the Maximin and Centroid design strategies, based on value of information criteria, are more efficient in determining points on the PF from the data than random selection, pure exploitation of the surrogate model prediction or pure exploration by maximum uncertainty from the learning model. Although the datasets varied in size and source, the Maximin algorithm showed superior performance across all the data sets, particularly when the accuracy of the machine learning model fits were not high, emphasizing that the design appears to be quite forgiving of relatively poor surrogate models.

## Introduction

Methods from data science are being increasingly applied to materials data to make predictions of new materials with targeted properties^1–10^. High throughput density functional calculations, for example, have been widely used to generate data in the tens of GigaBytes (e.g., in repositories such as materialsproject.org ^11^, Aflowlib^12^ and OQMD^13^), and then this data is analyzed to make predictions. In addition, there is growing interest in finding methods which efficiently guide the next experiments or calculations within an active learning feedback loop^14^. This approach is a departure from merely exhaustively computing in the search space of allowed materials, as most studies have undertaken. Feedback from the result of a computation or measurement can lead to a better materials selection strategy for the next computations or experiments. Here we integrate the feedback when multiple properties are involved along with uncertainty based statistical selection strategies in the materials design process.

Recently, we demonstrated how machine learning models in conjunction with optimization strategies, can guide the next experiments or calculations towards finding materials with desired single objectives or properties^15,16^. Using an adaptive learning paradigm based on active or reinforcement learning ideas from computer science, we showed how to iteratively select or recommend candidates for experiments or calculations and then update known training data with each new sample synthesized or computed to subsequently improve the search. New alloys^15^ and piezoelectric compositions^16^ with desired very low dissipation or phase boundary characteristics were found in this manner. Because of the vast search space and limited training data, the probability of finding these compounds by conventional trial and error approaches is exceedingly low.

**Figure 1:**
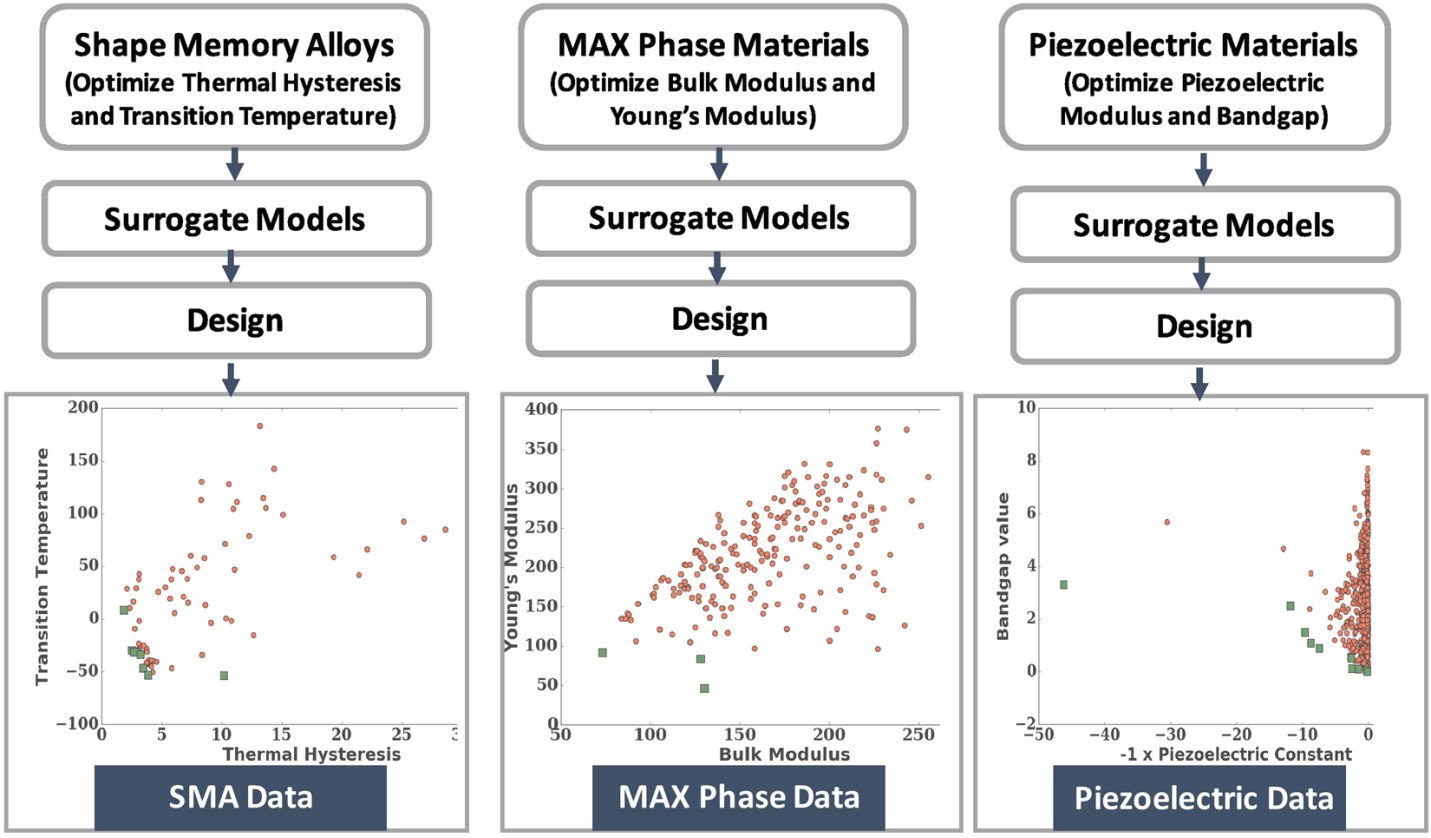
The scope of the multi-objective optimization in this work involving materials data sets for shape memory alloys, *M*_2_*AX* phases and piezoelectrics. The goal is to find the Pareto front, represented by the collection of green, square data points in the plots, for the data sets in as few iterations as possible using surrogate modeling and design. A subset from the full data set is available to begin the process. We compare the performance of different algorithms.

In contrast to finding materials with single optimal properties, it is usual when dealing with two or more properties, that is, objectives, to plot candidate materials on a so called Pareto plot, where the axes are the properties so that we can define a characteristic boundary on which lie materials where none of the objectives can be improved in value without degrading the other objective value. Such boundary points, the non-dominated data-points, define a Pareto front (PF) that represents the best trade-off between the objectives. Common examples of Pareto Fronts include the Ashby plots, which display two or more properties, such as Young’s modulus and density, for many materials or classes of materials^17,18^. Methods to estimate such fronts, especially if an exhaustive search is too tedious, have been studied and applied for some time^19–22^. We recently used Monte Carlo sampling methods, in conjunction with machine learning models, to obtain Pareto fronts for dielectric polymer data^23^. However, few studies have addressed how to guide experiments or calculations to recommend optimal points in as few measurements or calculations as possible, especially where the data sizes are relatively small. Our objective is to demonstrate how such design and multi-objective optimization methods perform on differing materials data sets of varying sizes to distill guidelines for future studies for accelerated discovery of unknown compounds. We will use surrogate models, defined as computationally cheaper models or “fits”, which can be parametric or non-parametric, learned from data and commonly used in statistics and engineering design to approximate complex mechanisms^24^. These have proved effective as a part of optimization algorithms for multi-objectives for nearly continuous PFs. But materials data often have a PF spanned by discrete points which can be located far away from each other. The goal of our design strategy is to find this unknown PF from initially known data with as few new measurements as possible (see Fig. 1). In the data sets we consider, the PF is known as all the data is known. But we will consider it to be unknown as we begin the design cycle and start to compute a sub-optimal front (sub PF) for the data. After a few design cycles, the sub PF will contain some of the points which are common to the optimal PF. The knowledge of the PF is used only as a stopping criterion for the design cycle, which is of course not possible in a real multi-objective design challenge when seeking an unknown compound. In the real scenario, the design process can be stopped either when a material with desired properties is found or when the budgeted resources have reached their limits.

We will use methods recently adapted for multi-objective problems based on single-objective, global response surface modeling (RSM), design of experiment (DOE) techniques and kriging, a data fitting procedure based on Gaussian processes^25,26^. These are being used in aerospace design to accelerate single-objective optimization approaches when expensive codes are involved^24^. We will use these developments to show how we can construct multi-objective Pareto plots for limited available data in materials science by accelerating the process of finding the PF for different classes of materials. The algorithms are based on maximizing the expected improvement *E*[*I*] in choosing the next candidate data point^27^, and we will study different choices for *E*[*I*]. The improvement *I* refers to the possible gain in the objectives in the next design cycle, and is calculated with respect to the materials in the current PF of already available or known data. We will study a purely experimental data set for shape memory alloys and two data sets of computationally derived data using density functional calculations. The experimental data set is for the thermal dissipation and martensitic transition temperatures for NiTi-based shape memory alloys containing Ni, Ti, Cu, Fe and Pd with almost 100 compounds. Previously this data set for thermal dissipation was compiled as a result of prediction, synthesis and characterization of new NiTi-based alloys with very low thermal dissipation^15^. The addition of transition temperatures to this high quality data set, constructed from measurements from one laboratory only, makes this ideal for our multi-objective study. One of our computational data sets is for the elastic properties of compounds belonging to the *M*_2_*AX* phases with hexagonal symmetry in which X atoms reside in the edge-connected M octahedral cages and the A atoms reside in slightly larger right prisms^28^. Over 240 chemical compositions have been exhaustively enumerated and their elastic moduli calculated from density functional theory. We consider the problem of finding compounds with the largest bulk and shear moduli; the single objective case was previously studied^28^. The final data set with over 700 compounds, compiled using the materialsproject.org database^11^, is for piezoelectrics where the aim is to find those materials with the maximum piezoelectric modulus and smallest band gaps, potentially important in finding new ferroelectric photovoltaics. Figure 1 shows the overall scope and the data sets for the materials problems studied in this work.

Our choice of experimental and computational data sets with varying sizes is guided by the need to find a robust strategy that works across the different types of data. Our objective is to compare the relative performance of the multi-objective methods on these material data sets in finding materials close to points on the Pareto front in as few iterations as possible. Our main finding is that the Maximin and Centroid based design strategies for materials discovery are more efficient than random selection, pure exploitation, in which the “best prediction” from the surrogate or learning model is used in finding points on the PF, and exploration strategies in which it is the prediction of the point with maximum variance or uncertainty from the learning model which determines points on the PF. The Maximin based design algorithm, which balances exploration and exploitation relative to the more exploratory Centroid strategy, performed better than both pure exploitation and pure exploration, especially if the training dataset is smaller. Although the datasets used in this work varied in size, fidelity and source, the Maximin optimization algorithm showed superior performance across these cases in which the accuracy of the machine learning regression model fits were too low to be considered reliable for predictions. Although we assume in this work that the Pareto front is known, our work provides the basis for choosing effective methods for guiding experiments, especially high throughput experiments with relatively fast turn around, or targeted simulations using computer codes, to iteratively find materials with multiple properties closest to the Pareto front. The work can also be extended to more than two objectives. After defining and discussing the concept of the Pareto front, in Sec. 2 we review the ideas underlying the value of information and basis for improvement in choosing the next “experiment” or data point, a key aspect of global optimization. We then describe the multi-objective strategies we employ and discuss their performance on our data sets in Sec. 3.

## Pareto front

**Figure 2:**
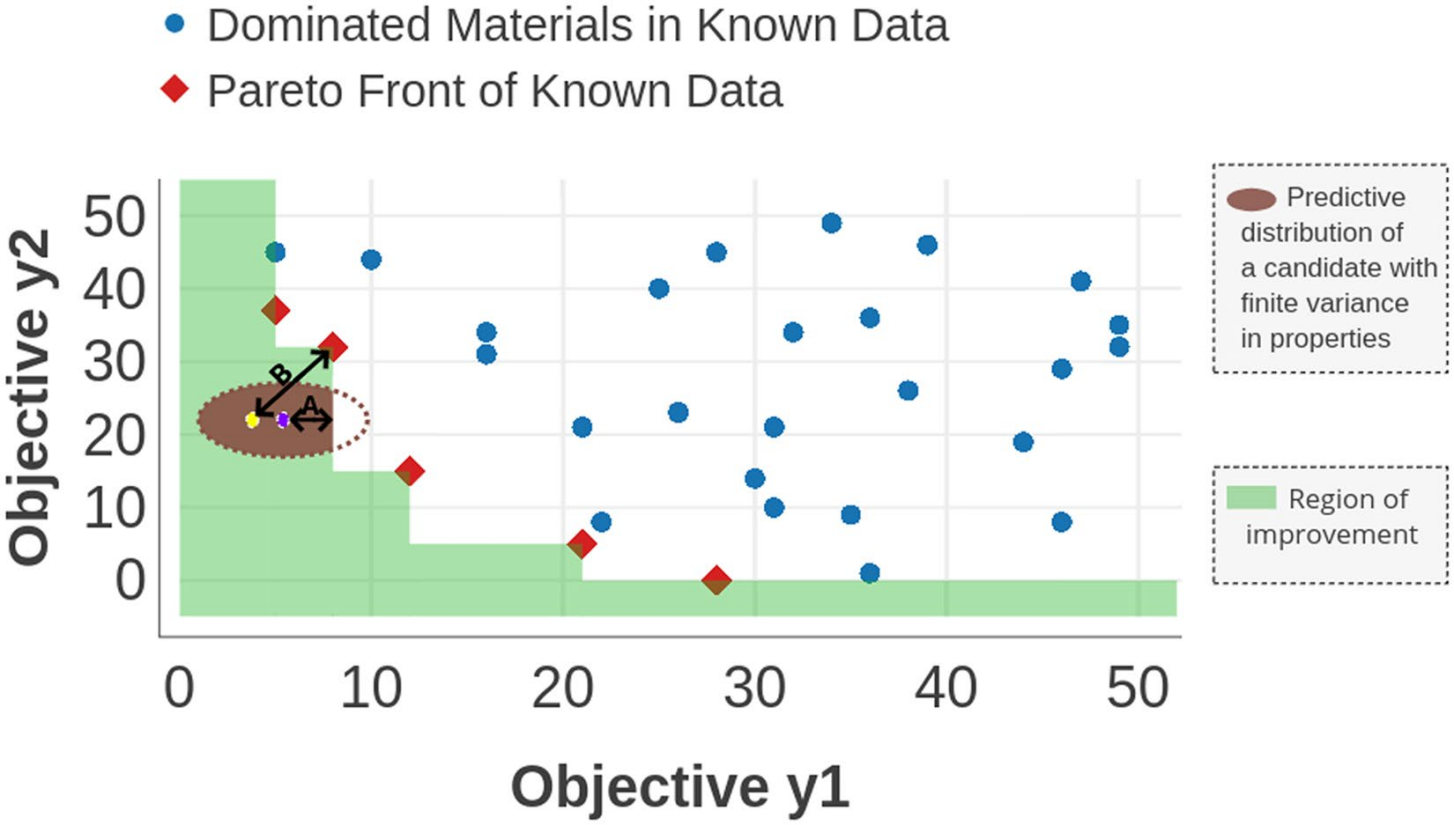
The figure depicts a schematic representation of data and its Pareto Front based on the assumption that both the properties are to be minimized. The PF will be convex towards the origin if all the properties were to be maximized. For a mixed problem with both minimization and maximization, the concaveness of the PF will be rotated by 90 degrees. The square points in red color represent the PF of data whereas the gray color dots are the points which are dominated by the PF. The region in white is the dominated region and the green shaded region is the region of improvement. Occurrence of a new material in the green shaded region could replace at least one existing PF point and thus lead to an improvement from the current PF. The brown shaded area corresponds to the predicted Gaussian distribution of one candidate material. The distribution can have different variations along axes because of the use of independent regression models to learn and predict each property. The violet point inside the brown shaded region represents the mean point of the entire predicted distribution of that particular material. The yellow point indicates the centroid of the predicted distribution lying inside the region of improvement. It is possible that the entire predicted distribution of some candidate material may lie inside the region of improvement. In that case, the mean of the entire distribution would coincide with the centroid. The distances A and B represent *L*_*maximin*_ and *L*_*centroid*_, respectively.

A Pareto front (PF) represents the data points which are not dominated by any other points in a data set. For example, consider an optimization problem where quantitative values of multiple properties are to be optimized, that is, either maximized or minimized among a set of materials. A particular material *M* is dominated if there exists another material which has more preferred quantitative values for all the considered properties than material *M*. It is highly unlikely in a real scenario that a single material has most preferred values for all the properties considered. A Pareto front for a multi-objective optimization problem is the analog of a data point with global minimum (maximum) value for a single objective minimization (maximization) problem. For *m* objectives or properties, if *y* = {*y*_1_(*x*), *y*_2_(*x*), *y*_3_(*x*), …, *y*_*m*_(*x*)} is the set of objectives for a material identified by a material descriptor (feature) vector *x* = (*x*_1_, *x*_2_, … *x*_*n*_), then we are interested in finding the *x* optimizing all objectives in *y*. In general, a unique solution satisfying all objectives does not exist, and we thus seek the set of optimal solutions on the Pareto front. Such solutions are based on the definition of dominance such that *x* is said to Pareto dominate *x*′ if ${y}_{i}(x)\leqslant {y}_{i}(x^{\prime} )$ for all *i* = 1, 2, …, *m* and *y*_*i*_(*x*) < *y*_*i*_(*x*′) for at least one *i* = 1, 2, …, *m*, that is, *x* is as good as *x*′ in all objectives and is strictly better in at least one. An *x* not dominated by any other is called Pareto optimal and the set of all Pareto optimal solutions constitutes the Pareto front. A PF plot with two objectives is shown in Fig. 2.

### Surrogate models and improvement criteria for multi-objective design

Surrogate models are widely used in the design community to represent expensive computational data in order to carry out optimization studies^25,26,29^. A fitted model becomes the basis for locating new and interesting combinations of features, which are then fed back into the code to update the surrogate model, and the whole process is repeated until the user runs out of resources or sufficiently improved designs are achieved. The update process tries to ensure that the model is reasonably accurate throughout the whole space, that is, there is “exploration” and that it also converges to the global minimum rapidly due to “exploitation”. Thus, there is competition amongst these goals in that to accurately learn the model we need to run our code (or perform experiments) in regions with little data and need to search in the most promising regions of the search space to exploit the solution. We have previously dealt with this problem^28^ using the concepts of probability of improvement and the expected value of improvement over the current best design in selecting the next calculation or measurement^27^. For a single objective, given a material property *y* dependent on features, also called as descriptors, *x*, machine learning allows us to estimate a function *f*(*x*) from the training data, such that $\hat{y}=f(x)$. However, in order to minimize the number of new materials that need to be experimentally tested, say, to find the material with the smallest *y*, we can choose a newly calculated design point *y*(*x*^*N*+1^) representing an improvement over the current best design, *f* ^*min*^(*x*) = *min*[*f* ^1^(*x*^(1)^), *f* ^2^(*x*^(2)^), … *f* ^*N*^(*x*^(*N*)^)], using *P*[*I*] and *E*[*I*], the probability and expected value of improvement. The improvement *I* is

1 $$I={f}^{min}(x)-\hat{y}({x}^{(N+\mathrm{1)}})$$

with

2 $$\begin{array}{rcl}P[I] & = & P[y({x}^{(N+\mathrm{1)}})\leqslant {f}^{min}(x)]\\  & = & {\int }_{-\infty }^{{f}^{min}(x)}\,\tfrac{1}{\sigma ({x}^{(N+\mathrm{1)}})\sqrt{(}2\pi )}exp(-\tfrac{{[\hat{y}-\mu ({x}^{(N+\mathrm{1)}})]}^{2}}{{\sigma }^{2}({x}^{(N+\mathrm{1)}})})\\  & = & {\rm{\Phi }}[\tfrac{{f}^{min}(x)-\mu ({x}^{(N+\mathrm{1)}})}{\sigma ({x}^{(N+\mathrm{1)}})}]\end{array}$$

The function Φ is the cumulant distribution function of the Gaussian integrands, and we have assumed that the new points are distributed according to a Gaussian distribution. Similarly, it can be shown that the expected improvement is

3 $$E[I]=[{f}^{min}(x)-\hat{y}({x}^{(N+\mathrm{1)}})]{\rm{\Phi }}[\tfrac{{f}^{min}(x)-\mu ({x}^{(N+\mathrm{1)}})}{\sigma ({x}^{(N+\mathrm{1)}})}]+\sigma \varphi [\tfrac{{f}^{min}(x)-\mu ({x}^{(N+\mathrm{1)}})}{\sigma ({x}^{(N+\mathrm{1)}})}],$$

where *ϕ* is the Gaussian probability density function. This design prescription is effective on a number of materials problems for single properties. We have applied it to experimentally find new NiTi based alloys with the smallest dissipation^15^ and shown how to minimize droop, the fall-off in the quantum yield as a function of number of quantum wells, in the design of Light Emitting Diodes (LEDs) using the industry code APSYS for semiconducting materials^30^.

The objective of experimental design is to optimally choose the next data point or sample predicted by the surrogate model (regressor) for synthesis, characterization or calculation. Efficient strategies become especially important when the costs of experiments or calculations are high and the objective becomes to minimize the number of such experiments or calculations. Our focus here is on the application to materials of the two-objective optimization problem. The green shaded region in Fig. 2 indicates the region where the occurrence of a candidate material after measurement would result in an improvement over the current front shown in blue dots. That means that the current subPareto front would be modified to include the newly measured material. The probability of improvement *P*[*I*] that the new point is an improvement over all existing points is the total probability of a candidate data-point integrated over the green shaded region in Fig. 2 and is given by

4 $${\rm{Probability}}\,{\rm{of}}\,{\rm{Improvement}},\,P[I]={\int }_{Shaded}\,\varphi ({y}_{1},{y}_{2})d{y}_{1}d{y}_{2},$$

where *y*_1_ and *y*_2_ are the objectives and *ϕ*(*y*_1_, *y*_2_) is the uncorrelated Gaussian probability distribution function formed from the mean and variance of *y*_1_ and *y*_2_ distributions with *ϕ*(*y*_1_, *y*_2_) = *ϕ*(*y*_1_)*ϕ*(*y*_2_). We have therefore assumed a Gaussian distribution for the predicted values with a mean and variance. Similarly, the equivalent two objective expected improvement *E*[*I*(*x*)] is the first moment of *I* of the joint probability distribution *ϕ*(*y*_1_, *y*_2_) over the green area in Fig. 2 about the current subPareto front. Geometrically, we can calculate *E*[*I*(*x*)] = *P*[*I*(*x*)]*L* in two ways depending on how the “length” L is evaluated: using the (a) Centroid or (b) Maximin approaches. We describe both and compare their relative performance in this work.

- Centroid approach to EI, referred to as EI-Centroid: *E*[*I*(*x*)] = *P*[*I*(*x*)]*L*, where $L=\sqrt{{({Y}_{1}(x)-{y}_{1}(x))}^{2}+{({Y}_{2}(x)-{y}_{2}(x))}^{2}}$, the distance between the centroid (*Y*_1_(*x*), *Y*_2_(*x*)) at the candidate data point, *x*, and closest point on the subPareto front, (*y*_1_(*x*), *y*_2_(*x*)). The centroid of the probability distribution for the candidate point in the green shaded region is calculated using
  5 $${Y}_{1}(x)={\int }_{Shaded}\,{y}_{1}\varphi ({y}_{1},{y}_{2})d{y}_{2}d{y}_{1}/P[I]$$
  Similarly for *Y*_2_(*x*).
- Maximin approach to EI, referred to as EI-maximin: Let the mean predicted values for a candidate material be (*μ*_1_, *μ*_2_). Then we define the distance *d*_*maximin*_ = *Max*_*i*_(*Min*(*p*_*i*1_ − *μ*_1_, *p*_*i*2_ − *μ*_2_), 0), where *P*_*i*_ = (*p*_*i*1_, *p*_*i*2_) and *P*_*i*_ ∈ *PF*. The maximin Expected Improvement is then *EI*_*maximin*_ = *d*_*maximin*_ × *P*[*I*(*x*)].

**Figure 3:**
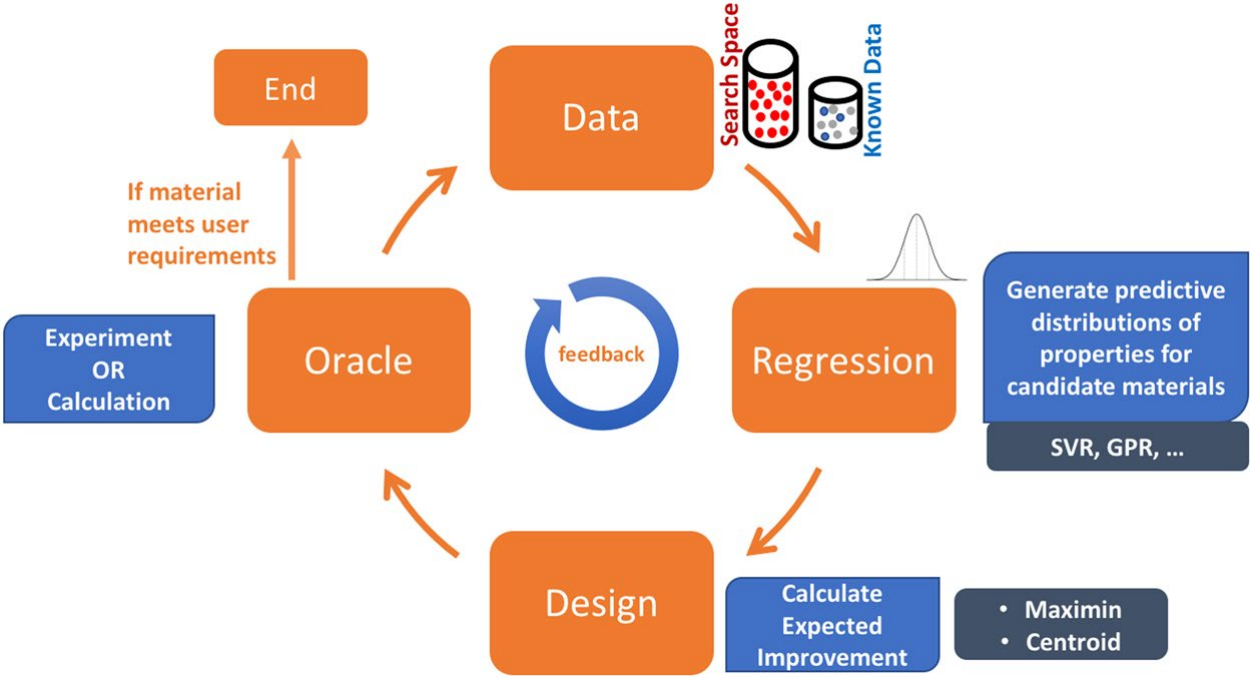
Design Flow. The design process begins with the prior training data, the set of materials with known values of their properties. A search space, the set of materials whose properties are not yet measured or calculated, the candidate data points in the design process, is then constructed. The next step is to build a regression model from the training data and then predict distributions for values of properties for each material in the search space. The finite distribution of each material is used to calculate the Expected Improvement, E(I). In this work we calculate E(I) using two approaches: Centroid-based and Maximin-based. The material with highest value of E(I) is chosen for measurement of its properties. The oracle represents either experimental measurements or high fidelity calculations of the material properties. If the newly measured material satisfies the user requirements, the design process is ended, otherwise, the new data is added to the training set for the next cycle. This adaptive design incorporates feedback from new measurements to increase the efficiency of subsequent design cycles.

Thus, for each candidate point in the region of improvement, EI-Centroid is calculated by taking the product of *P*[*I*] with the minimum distance between points on the known sub pareto front and centroid of the probability distribution within the region of improvement. The candidate point with the largest EI-Centroid is then the choice for the next measurement. EI-maximin is the product of *P*[*I*] and the maximum of the minimum distance of either of the means (*μ*_1_, *μ*_2_) of a particular candidate point from individual sub Pareto front points *p*_*i*_. The former considers improvement over the properties *y*_1_, *y*_2_ combined, whereas EI-maximin considers each property separately, takes the one which is smaller from a particular subPareto point, and then maximizes that amongst all the subPareto points. Both strategies select a data-point such that its measurement produces maximum modification to the sub Pareto front. We implemented both *EI*_*Centroid*_ and *EI*_*maximin*_ strategies and also compared them against (*i*) random selection, (*ii*) pure exploitation using only the mean values of predictions from machine learned model and finally (*iii*) pure exploration, where the selection is based on the magnitude of the variance for candidate points in the region of improvement. Our overarching design process is illustrated in Fig. 3.

### Regressors

**Figure 4:**
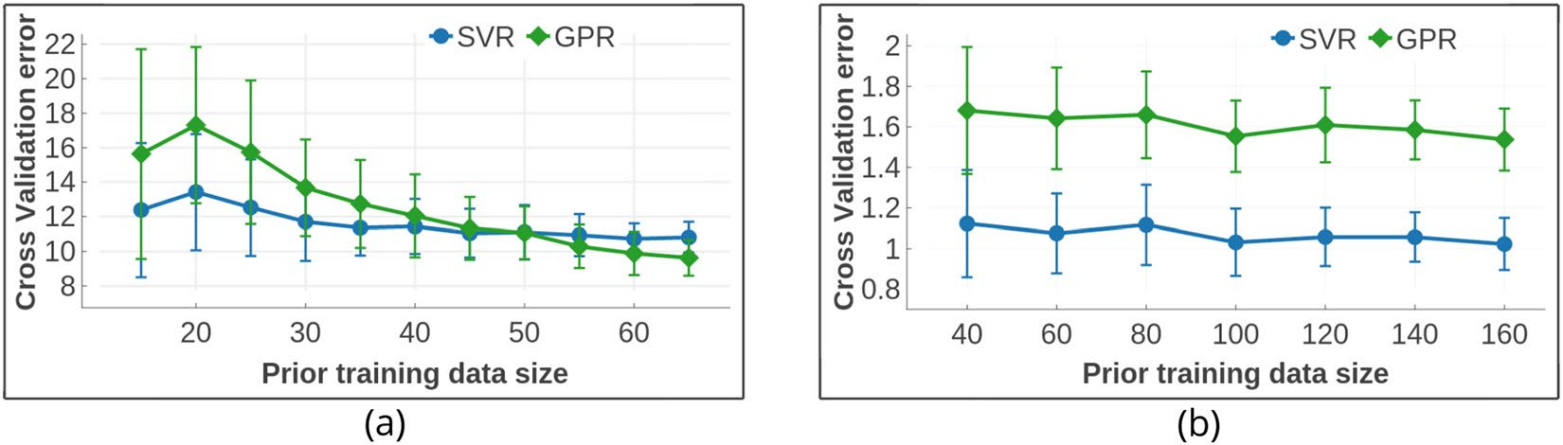
Performance of the regression models applied to (**a**) the shape memory alloy and (**b**) piezoelectric data sets. Gaussian Process Regression (GPR) and Support Vector Regression models were tested for their reliability with relatively small-size materials data. Reliability of regressors was measured in terms of their ten-fold cross validation scores for datasets with size above 20 and Leave One Out cross validation scores for smaller datasets. The size of training dataset is plotted on the horizontal axis and the average cross validation score is the ordinate. The regression models were cross validated fifty times for each training set-size for both datasets.

The surrogate models were built by fitting a mathematical function to the available data (training data). An estimate of the function *y* = *f*(*x*) from the data *x* for the surrogate model is provided by using regression schemes. The underlying assumption in evaluating the expected improvement *E*[*I*] is the Gaussian nature of the surface on which the data is distributed. This naturally leads to a Bayesian approach based on Gaussian process regression with a prior in terms of a mean function and covariance matrix, from which a posterior at a new point may be evaluated. We have tested both Gaussian Process Regression (GPR) model and Support Vector Regression^31^ (SVR) with Gaussian Radial Basis Function (RBF) kernel to compute the mean and variance for *y*. Upon fitting the function from training data, GPR produces both mean and variance for the predicted values of *y*; however, SVR does not generate a distribution for *y*. We therefore generated an ensemble of predictions for *y* and its variance (using bootstrapping) by training 5000 SVR models with subsets of the training data selected randomly and with replacement. Both GPR and SVR models were implemented using the Sci-Kit Learn^32^ Python library. The reliability of regression fits were measured using cross-validation. In an *n*-fold cross validation scheme, the training data is split into *n* equal sized subsets and each subset is predicted from a regression model trained with other *n* − 1 subsets. In this way, values are predicted for all subsets and compared with their real values. Using ten-fold cross validation for both models trained on Shape Memory Alloy data and Piezoelectric data, we find that the SVR model performs better as shown in Fig. 4. The reliability of the models was accessed using the *R*^2^ cross-validation score defined by equation (6)

6 $${\rm{Coefficient}}\,{\rm{of}}\,{\rm{determination}},\,{R}^{2}(y,\hat{y})=1-\frac{{\sum }_{i=0}^{{n}_{samples}-1}\,{({y}_{i}-\hat{y})}_{i}^{2}}{{\sum }_{i=0}^{{n}_{samples}-1}\,{({y}_{i}-{\bar{y}}_{i})}^{2}},$$

where *y* is the real data and $\hat{y}$ is the predicted data; *y*_*i*_ and ${\hat{y}}_{i}$ are the real and predicted values respectively for the *i*^*th*^ data-point and $\bar{y}$ is the mean of the real data *y*.

SVR has been shown to perform better than GPR in the case of elastic moduli data for the *M*_2_*AX* phases. That said, the fits to both SVR and GPR are not particularly good due to the small size of the training data. Thus, unlike large data problems, where machine learning tools are sufficiently reliable, the small data problems often encountered in material science require in addition a statistical design approach which can help to mitigate some of the shortfalls of the model.

### Algorithm

The optimization strategies discussed in the previous sections were tested and validated over the standard Binh-Korn^33^ test function data set and the three independent materials data-sets. The algorithm, in pseudo code form, given the data, surrogate and choice of *E*[*I*], is given below.

## Results

Our objective is to compare design strategies to find the optimal PFs for materials data-sets in as few design cycles as possible when the design process is initiated with a smaller subset of the data assumed to be initially known. We also assume that the optimal PFs are already known. We compared the sub-PF with the optimal PF after each measurement design cycle until the sub PF converged to the optimal PF. In general, the number of design cycles can be restricted by limiting the number of new measurements or when the sub-PF after a given number of measurements meets the requirements put on the materials properties by the researcher. Each dataset was divided into prior training data with known properties and materials in the search space with unknown values for the properties, respectively. The training data is updated after each design cycle till all the points in the optimal PF are found. We calculated the average number of design cycles needed to find the optimal PF for various sizes of prior training data. For statistics, the design process was repeated several times for each prior training data size selected randomly from the entire available dataset. The three materials datasets used in this work varied in total size, fidelity and source. The SMA data set is from experiments whereas the MAX phase and Piezoelectric data are the results of density functional theory (DFT) calculations. To bench mark our method, we first employed a discretized mathematical function, the Binh-Korn function test function, as a source of a relatively large amount of data.

**Algorithm 1:**
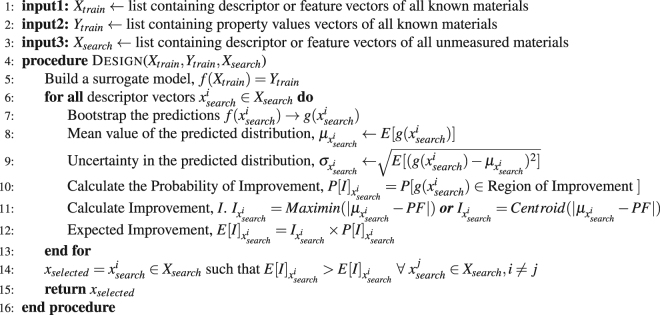
Multi-objective design algorithm

### Binh-Korn Function

The Binh-Korn test function problem is defined by:

Minimize {*f*_1_, *f*_2_} where,

$${f}_{1}(x,y)=4{x}^{2}+4{y}^{2};\quad {f}_{2}(x,y)={(x-\mathrm{5)}}^{2}+{(y-\mathrm{5)}}^{2}$$

Subject to the constraints,

$${(x-\mathrm{5)}}^{2}+{y}^{2}\le \mathrm{25;}\quad {(x-\mathrm{8)}}^{2}+{(y+\mathrm{3)}}^{2}\ge 7.7$$

**Figure 5:**
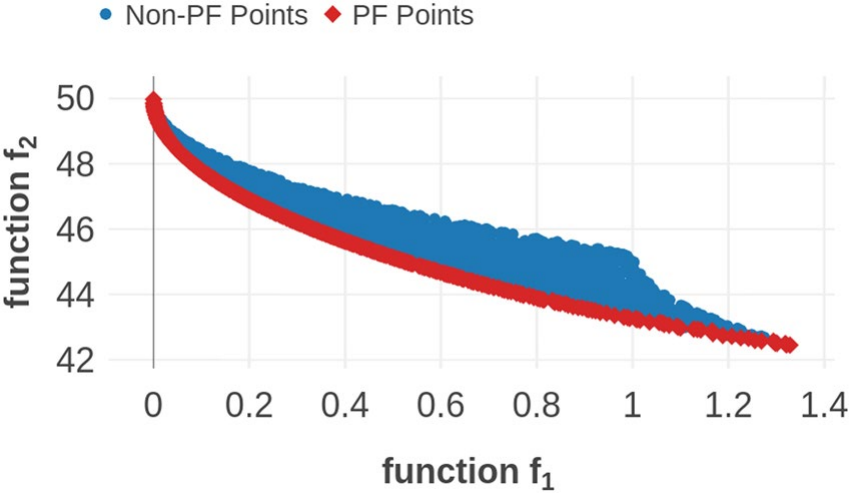
The Pareto Front of entire dataset is shown in red colored square dots. The points in blue color indicate the rest of the dataset dominated by the green colored PF points. In this dataset, the Maximin based design algorithm performed as well as the centroid-based algorithm.

From this function, a large dataset of size 70,471 was created within the search domain: 0 ≤ *x* ≤ 5 and 0 ≤ *y* ≤ 3, with *x*, *y* as features. The prior training set, assumed as a known set of points, was selected with twenty randomly selected data-points, intentionally excluding the optimal PF points. The size of the prior set was thus only 0.03% of the size of the total search-space. The goal was to find maximum number of data-points forming the optimal PF using MOO design strategies within a limited number of measurements. In total, 899 data points exist in the optimal PF, which is just nearly 1.2% of the total data-set generated. After 100 measurements, 69 points from PF were found using our design strategy, whereas an unbiased random selection strategy is expected to find only one PF point within that many measurements. This illustrates the optimization for *f*_1_, *f*_2_ is very effective in finding the PF points in the case of a limited number of measurements. The optimal PF of the entire dataset is shown in Fig. 5.

### Shape Memory Alloy (SMA) Data

The SMA data set is based on that developed in ref.^15^, where compounds belonging to the multicomponent NiTi-based family, *Ti*_50_*Ni*_1−*x*−*y*−*z*_*Cu*_*x*_*Fe*_*y*_*Pd*_*z*_, with the targeted property of low thermal hysteresis or dissipation were synthesized. The functionalities of SMAs, including the shape memory effect and superelasticity, arise from the reversible martensitic transformation between high temperature austenite and low temperature martensite phases. Heating and cooling across the martensitic transformation temperature results in hysteresis as the transformation temperatures do not coincide, giving rise to fatigue. Only the single objective, thermal hysteresis, was previously predicted and all the alloys constrained by $50-x-y-z\geqslant \mathrm{30 \% }$, $x\leqslant \mathrm{20 \% }$, $y\leqslant \mathrm{5 \% }$ and $z\leqslant \mathrm{20 \% }$ were synthesized by the same processing protocols in the same laboratory. With transition temperatures added to this data set of over 100 well characterized alloys, our goal is to find the compound in the data set which minimizes both the thermal hysteresis and the transition temperature. Each alloy is described in terms of one or more features representing aspects of structure, chemistry, bonding. There are many approaches to choosing features. Our choice was based on prior materials knowledge. It is known that the martensitic transition temperatures, which affect thermal hysteresis, are strongly correlated with the valence electron concentration and electron number per atom. In particular, the martensite and austenite start temperatures vary significantly when the valence electron concentration increases and show behavior that depends on the electron valence number/atom. Moreover, the thermal hysteresis is directly influenced by the atomic size of the alloying elements as the hysteresis increases with size at almost constant electron valence number. We thus used Zunger’s pseudopotential radii^34^, Pauling electronegativity, metallic radius, valence electron number, Clementi’s atomic radii^35^, and Pettifor chemical scale^36^ as features for the inference model^15^.

**Figure 6:**
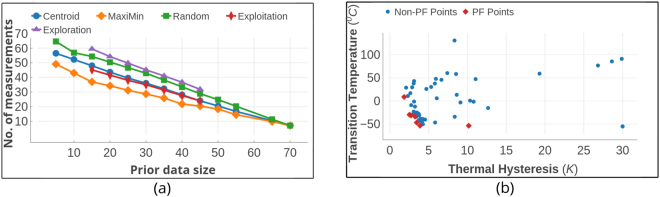
Shape Memory Alloy Data. (**a**) The size of the prior training dataset is plotted against the average number of design cycles required to find all the points in optimal PF. When the size of prior data is relatively small, the regression models deliver a less-reliable fit to the data. Thus, the Maximin design strategy in which the exploration and exploitation of data are more balanced, performs much better than all other methods. (**b**) Optimal Pareto Front. There are seven points in the optimal PF of this SMA data. The optimal PF is considered as unknown at the beginning of design process. Starting from a set of data-points which are considered as known, the goal is to find all the optimal PF points in as few design cycles as possible. The red colored square points form the optimal PF whereas each blue colored point is dominated by at least one point in the optimal PF.

**Figure 7:**
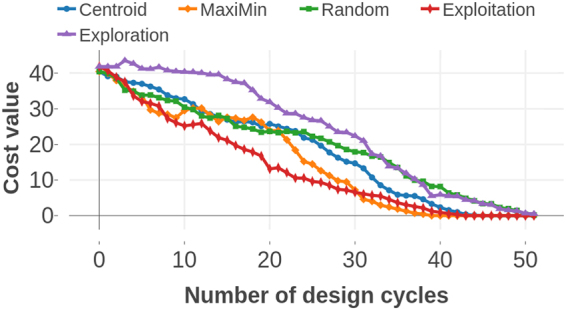
Cost value is defined as the average distance between data-points in Optimal PF and their individual closest neighbors in Sub PF. The plot indicates the statistically averaged cost against the number of design cycles performed. This particular graph was plotted for the SMA data beginning with 25 data points in prior training set and 52 data-points in search space. Use of Maximin design strategy converged the cost function to zero within a few measurements when compared with Centroid-based design and random selection approach.

As shown in Fig. 6(b), there are seven points in the optimal Pareto Front of this data set,. The design process was carried out using prior training data with varying sizes from 5 to 70. From Fig. 6(a), it is clear that employing MOO design strategies decreases the number of measurements required to find the optimal PF by nearly 20% compared to random selection. In addition, the MOO strategies reduced the computational effort by 40–45% compared to employing brute-force search to calculate all the candidate materials. The Centroid based design strategy and pure exploration perform similarly well; however, the Maximin approach shows superior performance compared to all other strategies, particularly if the prior datasets are smaller in size. In Fig. 7, we assess the convergence efficiency of the design strategies by plotting the cost function as a function of the number of design cycles. The cost function is defined as the average distance between the data points on the optimal front and their individual closest neighbors in the Sub-PF. The cost converges to zero within a few measurements compared to the Centroid strategy or random selection.

### Elastic Moduli Data for *M*_2_*AX* compounds (MAX)

**Figure 8:**
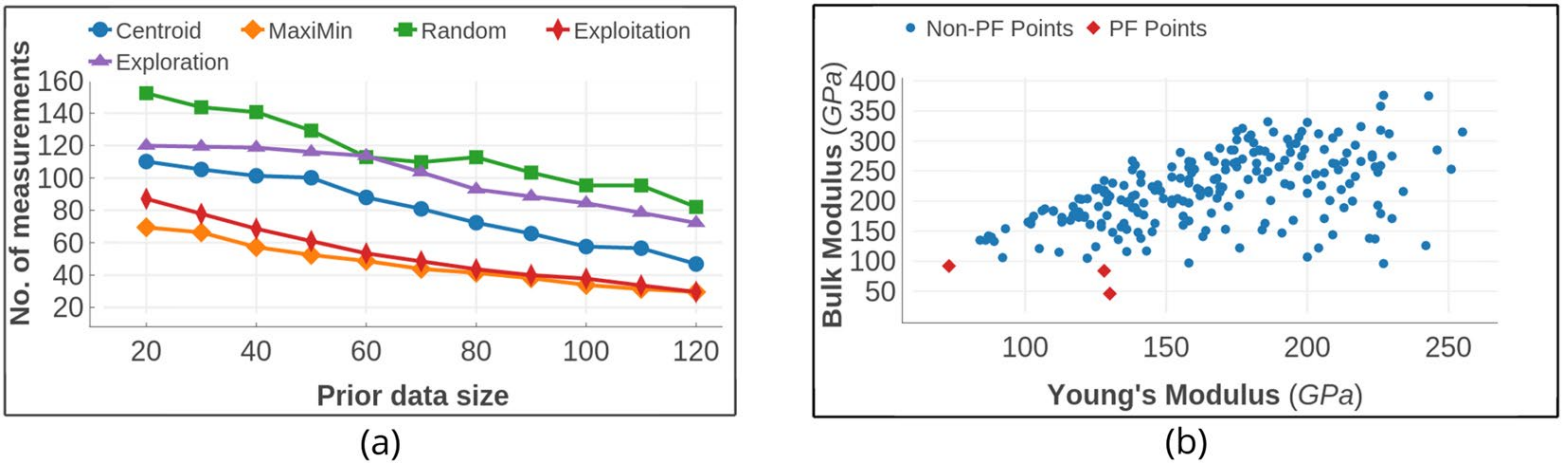
Elastic Moduli Data. (**a**) The size of prior training data is plotted against the average number of measurements required to find optimal PF. Maximin-based algorithm performed much better than pure exploitation, centroid-based design and random selection approaches. At the small initial training data, Maximin based design performs better than pure exploitation too. (**b**) Optimal Pareto Front. There are three materials in the optimal PF of this dataset of size 223. The red colored square points form the optimal PF while each blue colored point is dominated by at least one point in optimal PF.

This data set consists of computed elastic moduli values for 223 *M*_2_*AX* compounds and is a subset of 240 compounds calculated by Cover *et al*.^37^ using DFT calculations as implemented in the Vienna Ab initio Simulation Package (VASP)^38–41^ code using the projector-augmented wave (PAW)^42,43^ core potentials. We used orbital radii of the M, A, and X-atoms from the Waber-Cromer scale as features, which include the *s*-, *p*-, and *d*-orbital radii for M, while the *s*- and *p*-orbital radii were used for the A and X atoms^28^. This scale uses the self-consistent Dirac-Slater eigenfunctions and the radii correspond to the principal maxima in the charge-density distribution function for a specific orbital of a neutral atom. These features have been used previously and serve as good starting point because of the relationship between the electronic charge density and elastic response of materials. Factors such as elastic anisotropy that classify ductile from brittle materials have been shown to be related to the directional nature or the lack of chemical bonds formed between the *s*-, *p*-, *d*- or *f*-orbitals (near the Fermi level) of the nearest-neighbor atoms. The bulk and Young’s moduli were considered as the properties to be minimized and we performed the design process with prior data sizes ranging from 20 to 120. As shown in Fig. 8(b), three optimal PF points exist for this data-set. In Fig. 8(a), the best design strategy requires 55% fewer measurements than random selection and 65% fewer measurements than brute force to find all points in the optimal PF when the design process is initiated with a small prior training data set.

### Piezoelectric Materials Data

**Figure 9:**
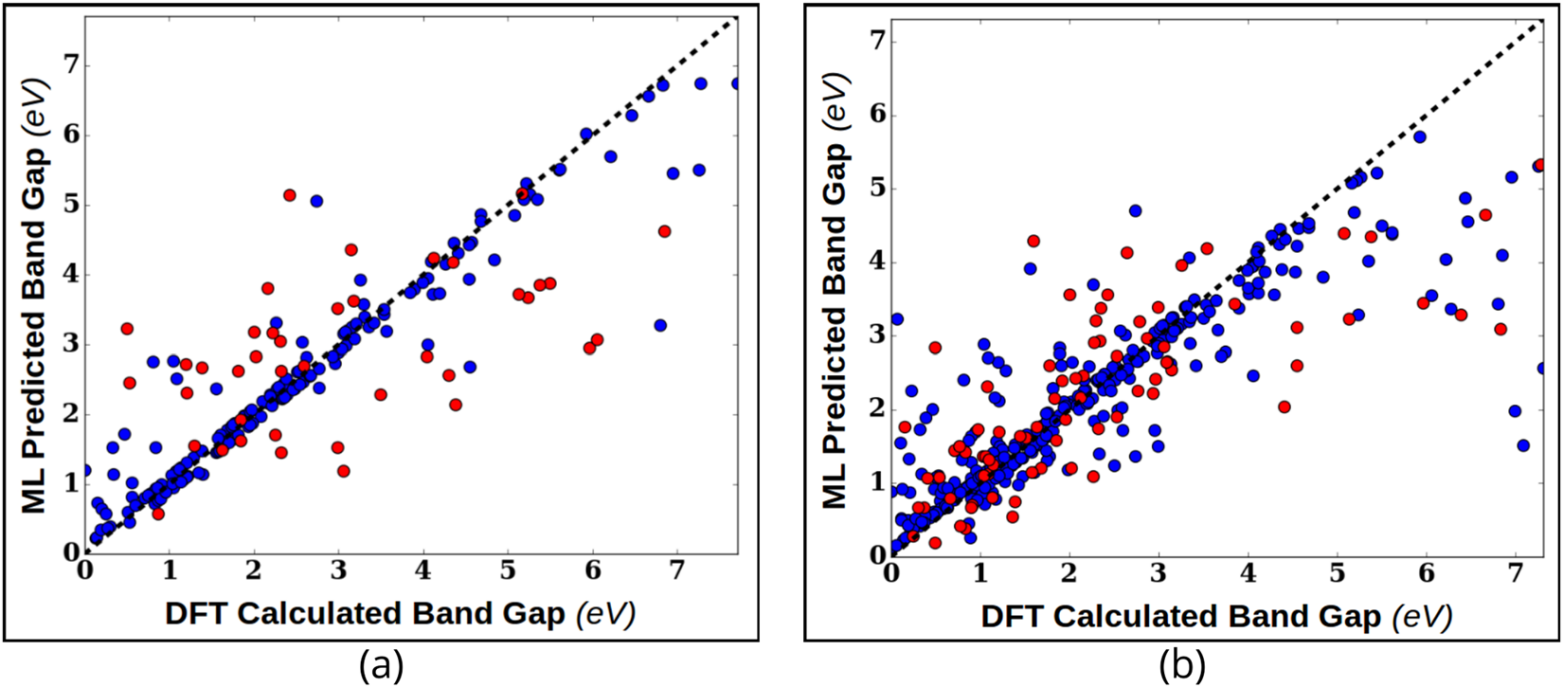
Surrogate model fit plots for bandgap values for the piezoelectric data. Real values are the data obtained through DFT calculations. The fits to the other datasets were similar. The horizontal and vertical axes span the real and predicted values of data-points, respectively. The design process is initiated with 200 training data-points. Blue points indicate the training data and red points correspond to the test data. The plots were taken randomly from one of the many design steps we carried out to analyze the design performance statistically. Since the surrogate model parameter tuning in these design processes was automated, there is a certain amount of over-fitting to the data. However, this can be avoided during a design problem for an unknown compound by tuning the surrogate model parameters carefully, (**a**) the model fit for the first design cycle with 200 training data points, (**b**) fit for the 200^*th*^ design cycle. After 200 cycles, the size of the training data increased to 400. This plot emphasizes that the design does not necessarily require a very good surrogate model for acceptable performance.

**Figure 10:**
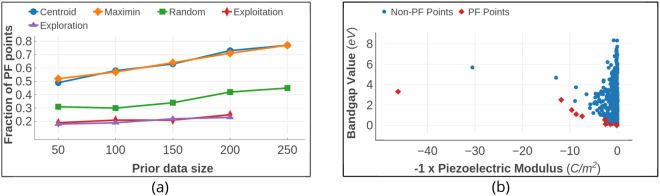
Piezoelectric Data from materialsproject.org. (**a**) The size of prior training data is plotted against the averaged fraction of data-points from optimal pareto-front found after 200 measurements. In this dataset, which is the largest in size, the design methods were efficient than the pure exploration/exploitation strategies. Owing to a large feature set size and small initial training data compared to the search space, the regression models cannot fit the data with sufficient reliability for predictions. (**b**) Optimal Pareto Front. Piezoelectric dataset contains data of 704 materials of which 11 form the PF. The red colored square points form the optimal PF while each blue colored point is dominated by at least one point in the PF.

This data-set was created through high throughput DFT-based *ab initio* computations^44^ and is archived in materialsproject.org ^11^. From it, we extracted the data for materials with computed values of band gaps and maximum piezoelectric longitudinal moduli using the Pymatgen^45^ Python package. In this data, the optimization objectives are to minimize the value of the bandgap and maximize the maximum piezoelectric modulus of the compounds. The piezoelectric property corresponds to the maximum attainable absolute value of the longitudinal piezoelectric modulus of the crystal in any direction. As the direction of the electric field is varied, it is the measured maximum response of the crystal over all possible directions. Ionic radii, Volume, Density, Electronegativity and Crystal point group were selected as dependent features after theoretical analysis of various structural and thermodynamic characteristics. Some of these features were directly available in the materialsproject.org while others, such as ionic radii, were calculated using Pymatgen. The full dataset had information of 941 piezoelectric materials. But for our work, it was reduced to 704 materials since the ionic radii of some materials in original set were not reliably resolved through Pymatgen. Even then, with 704 data points, the piezoelectric data is larger in size than aforementioned SMA and MAX data sets. Considering the relatively large size of the data-set, the maximum number of design cycles was limited to 200. Figure 9 shows diagonal plots illustrating the quality of the surrogate SVR models during the initial and final design cycles in which the design process was initiated with a training data size of 200. At the end of the design process with 200 cycles, the training data size increased by 200 to a total of 400 data-points. With each design cycle, more data points are added in the less explored areas of the feature space. Although the quality of the model fits is variable, the design is quite forgiving of a poorly fitting model and leads to acceptable performance. We measured the average number of optimal PF points found within this limited number of cycles. The dataset and optimal PF are shown in Fig. 10(b). The fraction of Pareto front points found after the limited number of measurements is used to compare the MOO design strategies with random selection. As shown in Fig. 10(a), both design strategies performed equally well and are more efficient than random selection. More than half of the Pareto-frontal points were found within the first 50 measurements.

## Discussion

The results presented for each dataset indicate that the Maximin based design strategy for materials discovery is more efficient than the Centroid strategy, random selection, pure exploitation or exploration, or just a brute force search to find materials on the PF in as few cycles as possible. It balances exploration and exploitation if the training dataset is significantly smaller than the search space. In the informatics based design approach, we are particularly concerned about such data-deficient situations where the predictive power of regression models is accompanied by large uncertainties due to large cross validation errors. For Maximin, the exploration part of the design algorithm enters through the probability of improvement *P*[*I*] and the “distance” *L*, which is dependent on the means, brings in the exploitation aspect. The algorithm performed well across all the data sets in which the accuracy of the machine learning regression model fits was too low to be considered reliable for predictions. Nevertheless, the optimal Pareto points could be determined within a few design cycles. This highlights an aspect of design that is increasingly becoming apparent on a number of materials problems and data sets^14,15,28^; that is, the design is quite forgiving of a poor surrogate model. The interplay between the two needs to be further explored and understood. The performance plots illustrate that the pure exploration strategy is less efficient than random selection, because in pure exploration the candidate material with the highest uncertainty is chosen for the next experiment. This means that the exploration algorithm entirely ignores the predicted values of properties and forces the design cycle to select a material which is most isolated from the known data. Pure exploitation performs as well as the Centroid based design. In the Centroid based design, the exploration-exploitation balance is tilted towards exploration if the centroid is far from the mean. It is important to consider the scale of the data associated with each of the properties: While *P*[*I*] is a dimensionless quantity, independent of the magnitude of the values of the objectives, the Expected Improvement quantifies the improvement and is a dimensional quantity. The *E*[*I*] is biased towards the objective property with larger magnitudes. Thus, this bias is avoided by normalizing the property values of known data-sets before each design cycle.

## Electronic supplementary material

Supplementary Dataset 1
